# A preoperative nomogram incorporating semantic MRI features for predicting breast cancer recurrence: a propensity score-matched cohort study

**DOI:** 10.3389/fonc.2026.1886709

**Published:** 2026-09-24

**Authors:** Cici Zhang, Yuchao Xiong, Chunpan Zhu, Shuang Wen, Jun Bu

**Affiliations:** 1 Department of Radiology, Guangzhou Red Cross Hospital, Guangzhou, China; 2 Department of Radiology, Dongguan Hospital of Traditional Chinese Medicine, Dongguan, China; 3 Department of Radiology, Guangzhou Chest Hospital Affiliated to Guangdong Pharmaceutical University, Guangzhou, China

**Keywords:** breast cancer, recurrence, MRI, nomogram, risk

## Abstract

**Background:**

Accurate preoperative assessment for postoperative recurrence risk remains challenging in breast cancer (BC). This study aimed to develop and validate a nomogram based on routinely available preoperative semantic MRI features for predicting postoperative recurrence in BC, using propensity score matching (PSM) to reduce confounding.

**Methods:**

A retrospective analysis was conducted in 395 patients with primary BC who underwent curative surgery and preoperative breast MRI between January 2010 and December 2023. MRI features were independently assessed by two radiologists blinded to clinical outcomes. PSM was applied to balance age, family history of BC, and type of surgery. After PSM (1:2 ratio), 204 patients (68 with recurrence and 136 without) were included for analysis. Univariate logistic regression identified candidate predictors (*P* < 0.05). Given the relatively low events-per-variable (EPV = 4.9, 68 events/14 candidate predictors), the Enter method was utilized for multivariate logistic regression based on clinical relevance to minimize the risk of overfitting. A nomogram was constructed and internally validated using bootstrap resampling (1000 iterations) with optimism correction.

**Results:**

Multivariate analysis identified three predictors of recurrence: adjacent vessel sign, tumor size >5 cm, and axillary lymph node metastasis. The nomogram demonstrated good discrimination, with areas under the receiver operating characteristic curve of 0.809 in the training cohort and 0.797 in the validation cohort. Calibration was satisfactory (slope = 1.000, intercept = −0.000), with mean absolute errors of 0.087 in the training cohort and 0.070 in the validation cohort. Decision curve analysis confirmed clinical utility across threshold probabilities of approximately 10% to 70%.

**Conclusions:**

The preoperative MRI-based nomogram demonstrated good predictive performance and satisfactory calibration in this internally validated cohort, suggesting its potential as a tool for individualized risk stratification prior to treatment. However, as this was a single-center study with internal validation only, external validation in independent multicenter cohorts is required before clinical implementation.

## Introduction

The latest global cancer statistics indicate that among women, breast cancer (BC) has surpassed lung cancer as the leading cause of cancer-related incidence and has become a leading cause of cancer-related mortality worldwide (1). In China, BC represents a major public health burden for women, with an estimated 3,246,625 new cases and 1,699,066 deaths reported in 2024 (2). Although advances in screening and treatment have improved overall prognosis, postoperative recurrence remains a major cause of treatment failure. In particular, patients with high-risk BC face substantially elevated recurrence risks; among patients with hormone receptor-positive, HER2-negative disease, the 5-year and 10-year distant recurrence risks can reach 20.1% and 31.4%, respectively (3). Recurrent BC is often more challenging to manage and is associated with a poorer prognosis, as definitive diagnosis typically requires confirmation via imaging, laboratory tests, or pathological examination, which inevitably delays the initiation of therapeutic intervention until metastasis is documented. Therefore, accurate identification of patients at high risk of recurrence before definitive treatment is critical to enable timely intervention while avoiding unnecessary overtreatment in those at low risk.

Emerging biomarkers, such as circulating tumor DNA (ctDNA), have shown promise for detecting minimal residual disease and predicting recurrence (4); however, their routine clinical adoption remains limited by high costs, technical complexity, prolonged turnaround times, and concerns over assay sensitivity and specificity. In contrast, dynamic contrast-enhanced magnetic resonance imaging (DCE-MRI) is routinely performed in clinical practice for tumor staging, assessment of extent of disease, and treatment planning (5). DCE-MRI provides both qualitative morphological information and quantitative perfusion-related parameters, offering a noninvasive means to characterize tumor biology and heterogeneity (6). Previous studies have investigated the association between MRI features and BC recurrence, focusing on two complementary categories of imaging biomarkers (7–11). Quantitative parameters, such as the apparent diffusion coefficient and time to peak enhancement, have been linked to increased recurrence risk (7); however, they are subject to substantial inter-scanner and inter-center variability, which limits their generalizability across different institutions. Qualitative semantic features, including subcutaneous edema, peritumoral edema, tumor size, tumor necrosis, and rim enhancement, have also been associated with recurrence in various BC subtypes and patient populations (7–10). Nevertheless, findings across studies remain inconsistent, many investigations are constrained by relatively small sample sizes, a low number of recurrence events, and inadequate adjustment for known prognostic factors such as age, family history, and type of surgery (11).

To better elucidate the prognostic value of MRI features, larger sample sizes and rigorous adjustment for confounders are warranted. Propensity score matching (PSM) provides a valid alternative to control for confounding in retrospective settings. PSM has been increasingly applied to reduce confounding; for instance, Zheng et al. used a 1:2 PSM approach to compare prognosis between neoadjuvant and adjuvant chemotherapy in patients with luminal BC (12). In the present study, PSM was similarly employed to minimize potential confounding and balance key baseline covariates, including age, family history of BC, and type of surgery, between patients with and without recurrence. After matching, balanced cohorts were constructed to enable more robust comparisons. The present study focuses on semantic MRI features that can be visually assessed on routine clinical images without additional computational tools. Using these features, a preoperative MRI-based nomogram was developed to predict postoperative recurrence in BC, to enables early identification of high-risk patients prior to definitive treatment and to informs intensified surveillance decisions, thereby providing evidence-based support for personalized treatment strategies.

## Materials and methods

### Study design and reporting guidelines

The study was designed and reported in accordance with the Strengthening the Reporting of Observational Studies in Epidemiology (STROBE) statement (13) and the Transparent Reporting of a multivariable prediction model for Individual Prognosis Or Diagnosis (TRIPOD) guideline (14). The study protocol was approved by the Institutional Review Board of our hospital (Approval No: 2024-450-01), and individual informed consent was waived for the retrospective analysis.

### Patient selection

This retrospective study included patients with histologically confirmed BC who underwent surgery at our institution between January 2010 and December 2023. The initial cohort comprised 567 patients with clinical stage I-III BC. All patients received multimodal treatment protocols tailored to their individual condition and the tumor’s clinicopathological and molecular characteristics.

Inclusion criteria were as follows: (1) histologically confirmed invasive breast carcinoma based on surgical specimens; (2) availability of complete preoperative breast MRI scans performed within one week before surgery, with no prior chemotherapy, biopsy, or other therapeutic intervention administered before the MRI; and (3) availability of complete clinical and follow-up data. Patients with bilateral breast cancer were included; in such cases, the primary tumor was defined as the lesion with the higher T stage.

Exclusion criteria were as follows: (1) pure ductal carcinoma *in situ* (DCIS); (2) severe motion artifacts or poor image quality on MRI that precluded reliable evaluation; (3) a history of, or concurrent diagnosis of, another malignant tumor; and (4) core needle biopsy performed immediately prior to MRI, due to biopsy-induced tissue changes (hemorrhage, edema, and inflammation) that may alter MRI signal characteristics and confound interpretation of native tumor morphology and enhancement patterns.

### Clinicopathological data and treatment

Comprehensive clinicopathological data were retrospectively extracted from the institutional electronic medical records. The variables collected included age, family history of BC, tumor stage, histologic grade, molecular subtype, and lymphovascular invasion. Molecular subtypes were classified based on immunohistochemistry (IHC) results for estrogen receptor (ER), progesterone receptor (PR), HER2, and Ki-67, in accordance with the 2013 St. Gallen International Breast Cancer Conference consensus. For tumors with HER2 IHC 2+, fluorescence *in situ* hybridization (FISH) was performed to determine HER2 amplification status.

### Postoperative follow-up

All patients were enrolled in a standardized postoperative surveillance program. Follow-up typically consisted of regular clinical examinations and radiological imaging. The imaging modalities included breast MRI, CT of the chest, abdomen, and pelvis, brain or spinal MRI, and bone scans, as clinically indicated. Suspicious findings were confirmed by biopsy as clinically indicated. The surveillance aimed to detect locoregional recurrence (defined as cancer recurrence in the ipsilateral breast, chest wall or regional lymph nodes) and distant metastasis (defined as disease spread to distant organs).

### Image acquisition and analysis

All breast DCE-MRI examinations were performed on a single 1.5-T scanner (Magnetom Avanto, Siemens Healthineers, Erlangen, Germany) using a standardized protocol throughout the entire study period. The use of a single scanner and standardized acquisition protocol minimized technical variability, ensuring consistency in image quality and feature assessment across all patients. The imaging protocol comprised axial T1-weighted sequences (repetition time/echo time (TR/TE): 8.6/4.7 ms), axial and coronal fat-suppressed T2-weighted imaging (TR/TE: 5600/59 ms), and a dynamic series. The dynamic series included one pre-contrast and five post-contrast bilateral acquisitions using fat-suppressed T1-weighted imaging (TR/TE: 7.72/2.53 ms; flip angle: 15°; matrix: 340 × 340; slice thickness: 3 mm). A standard dose of gadolinium-based contrast agent (gadobutrol, Gadovist, Bayer Schering Pharma, Berlin, Germany; 0.2 mmol/kg body weight) was administered intravenously.

All MRI features were evaluated independently by two breast radiologists (Reader 1 and Reader 2, with 7 and 9 years of experience in breast imaging, respectively), who were blinded to clinical outcomes. Interobserver agreement was assessed on the full cohort of 395 patients. Kappa coefficient (κ) was calculated for each categorical MRI feature. For any disagreement between the two readers, a consensus reading was performed during a joint session.

Image interpretation adhered to the 2013 Breast Imaging Reporting and Data System (BI-RADS) MRI lexicon. The following features were evaluated and defined as specified below:

(1) Amount of fibroglandular tissue (FGT): categorized as almost entirely fatty, scattered, heterogeneous, or extreme; (2) Background parenchymal enhancement (BPE): categorized as non/minimal, mild, moderate, or marked; (3) Tumor size: defined as the maximum diameter on the axial post-contrast T1-weighted image, with the size of the largest lesion recorded for multifocal disease; (4) Tumor type: classified as a mass; multifocal lesions (defined as those within the same breast quadrant or less than four cm away from the index lesion); or non-mass enhancement; (5) Tumor shape: classified as regular (round or oval) or irregular; (6) Tumor margin: classified as circumscribed or not circumscribed (including indistinct, angular, or spiculated margins); (7) Peritumoral oedema: defined as a high signal surrounding the tumor on fat-suppressed T2-weighted imaging; (8) Adjacent vessel sign: defined as an enhancing vessel entering or directly contacting the tumor margin on subtraction images; (9) Duct dilatation: recorded as present or absent; (10) Peripheral tissue invasion: defined as tumor invasion of the skin, chest wall, or pectoralis major muscle; (11) Nipple-areola complex invasion: recorded as present or absent. (12) Enhancement pattern: classified as homogeneous or heterogeneous enhancement; (13) Time-signal intensity curve (TIC) type: classified as persistent, plateau, or washout; and (14) Axillary lymph node metastasis (ALNM): defined as a lymph node that demonstrated a rounded or irregular shape, a short-axis diameter ≥1 cm, loss of the fatty hilum, or heterogeneous enhancement.

### Statistical analysis

All statistical analyses were performed using SPSS software (version 26.0; IBM Corp, Armonk, NY, USA) and R software (version 4.2.0; R Foundation for Statistical Computing, Vienna, Austria). A two-sided *P* value <0.05 was considered statistically significant. Categorical variables were summarized as frequencies and percentages and compared using the chi-square test or Fisher’s exact test, as appropriate. Continuous variables were presented as mean ± standard deviation (SD) and compared using the independent samples t-test when normally distributed; otherwise, non-parametric tests were applied. Tumor size was grouped into two categories (≤5 cm and >5 cm).

PSM was performed using a 1:2 nearest-neighbor matching algorithm with a caliper width of 0.2 times the standard deviation of the logit-transformed propensity score. Age, family history of BC, and type of surgery were chosen as matching variables, given the limited number of recurrence events and their clinical relevance as intrinsic host factors (age and family history) or treatment-related determinants of recurrence risk (type of surgery). Standardized mean differences (SMD) were calculated for all available baseline characteristics before and after matching, with SMD < 0.1 considered indicative of adequate balance.

Following PSM, all eligible patients were randomly divided into a training set and a validation set in a 7:3 ratio. The training set was used to develop the prediction model, while the validation set was used to evaluate generalizability. Potential predictors of postoperative recurrence were initially screened using univariate logistic regression analysis. Variables with *P* value <0.05 were subsequently entered into a multivariate logistic regression model using the Enter method to identify independent predictors. Additionally, to account for residual imbalance in type of surgery after PSM, this variable was forced into the multivariable model as a covariate. The events-per-variable (EPV) ratio was calculated to assess the reliability of the model.

A nomogram was then constructed based on the identified independent predictors. Model performance was assessed using the area under the receiver operating characteristic curve (AUC). To evaluate model robustness and correct for potential overfitting, bootstrap resampling with 1,000 iterations was performed separately within both the training set and the validation set. In each iteration, a bootstrap sample of the same size as the respective dataset was drawn with replacement. The model was refit using the bootstrap sample, and its performance was evaluated in both the bootstrap and original samples. The difference between these two performance estimates was calculated as the apparent optimism. The average optimism across all 1,000 iterations was then subtracted from the apparent performance to derive the optimism-corrected performance. Calibration was evaluated using calibration plots, with the calibration slope and intercept calculated from the regression of observed outcomes on predicted probabilities. The mean absolute error (MAE) between predicted and observed probabilities was also calculated, with an MAE < 0.10 considered indicating good calibration. The Hosmer-Lemeshow goodness-of-fit test was additionally performed to assess calibration, with P > 0.05 indicating adequate fit. Additionally, Decision curve analysis (DCA) was also performed to evaluate the net clinical benefit of the nomogram across a range of threshold probabilities.

## Results

### Characteristics of eligible patients

The study flowchart is presented in **Figure 1**. After applying the exclusion criteria, 172 patients were excluded: 62 were lost to follow-up, 44 had incomplete clinical data, and 66 had received neoadjuvant chemotherapy or core needle biopsy prior to MRI. A total of 395 eligible patients (68 with recurrence and 327 without recurrence) were included in the final analysis. Baseline clinical characteristics of the recurrence and non-recurrence groups are summarized in **Table 1**. There were no significant differences between the two groups in terms of age, menopausal status, family history of BC, lymphovascular invasion, histologic grade, molecular subtype, or surgery approach (all P>0.05).

**Figure 1:**
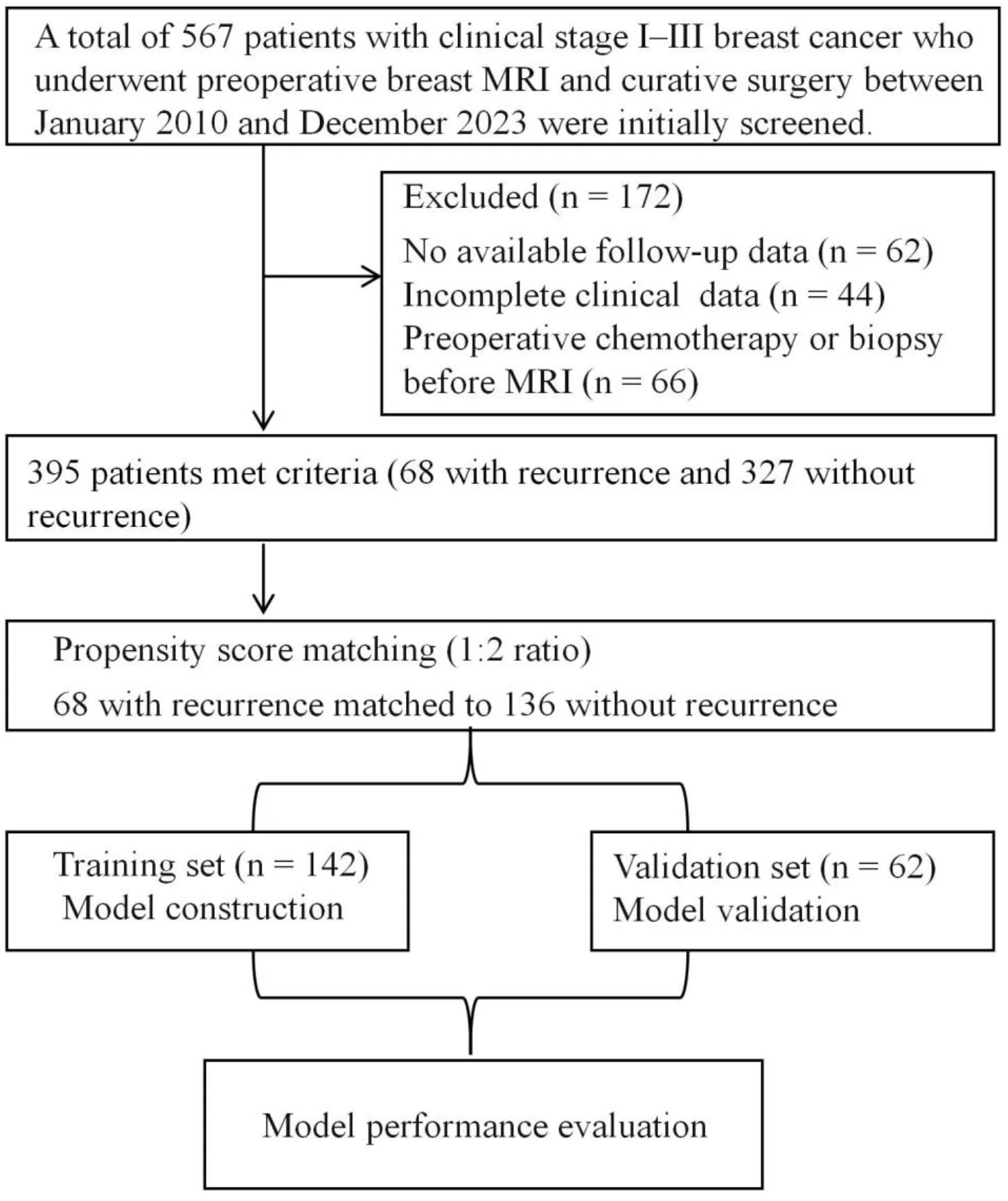
Study flowchart.

**Table 1:** Comparison of characteristics between recurrence and no-recurrence groups.

Characteristics	Recurrence (n=68)	No-recurrence (n=327)	T/χ^2^	*P*
Age (years), mean ± SD	58.22 ± 13.10	57.46 ± 11.97	-0.467	0.641
Menopausal status, n (%)			0.003	0.953
Premenopausal	22 (32.4)	107(32.7)		
Postmenopausal	46 (67.6)	220 (67.3)		
Family history of breast cancer, n (%)			1.543	0.214
No	7 (10.3)	20(6.1)		
Yes	61(89.7)	307(93.9)		
Lymphovascular invasion, n (%)			3.332	0.068
No	39(57.3)	225(68.8)		
Yes	29(42.7)	102(31.2)		
Histologic grade, n (%)			1.137	0.286
I/II	36(52.9)	196(59.9)		
III	32(47.1)	131(40.1)		
Molecular subtype, n (%)			2.844	0.241
Luminal	50(73.5)	250(76.5)		
HER-2 enriched	10(14.7)	57(17.4)		
Triple negative	8(11.8)	20(6.1)		
Surgery type, n (%)			0.582	0.446
Breast-conserving	3(4.4)	26(7.9)		
Mastectomy	65(95.6)	301(92.1)		

### Characteristics of recurrence

The median follow-up duration for the entire cohort was 59 months (interquartile range: 38–82 months). For patients who remained recurrence-free throughout the study period, the minimum follow-up was 18 months, with censoring at the date of last clinical contact or death.

68 patients developed recurrence. Of these, 22 patients experienced locoregional recurrence, 19 had single-organ metastasis, and 27 presented with multiple organ metastases. The most frequent sites of distant metastasis were bone, lung, and/or pleura (37 patients, 54.41%), followed by the liver (20 patients, 29.41%). Metastases to other sites, including the brain, meninges, spleen, and lymph nodes, were observed. The distribution of recurrent and metastatic sites is shown in an UpSet plot in **Figure 2**.

**Figure 2:**
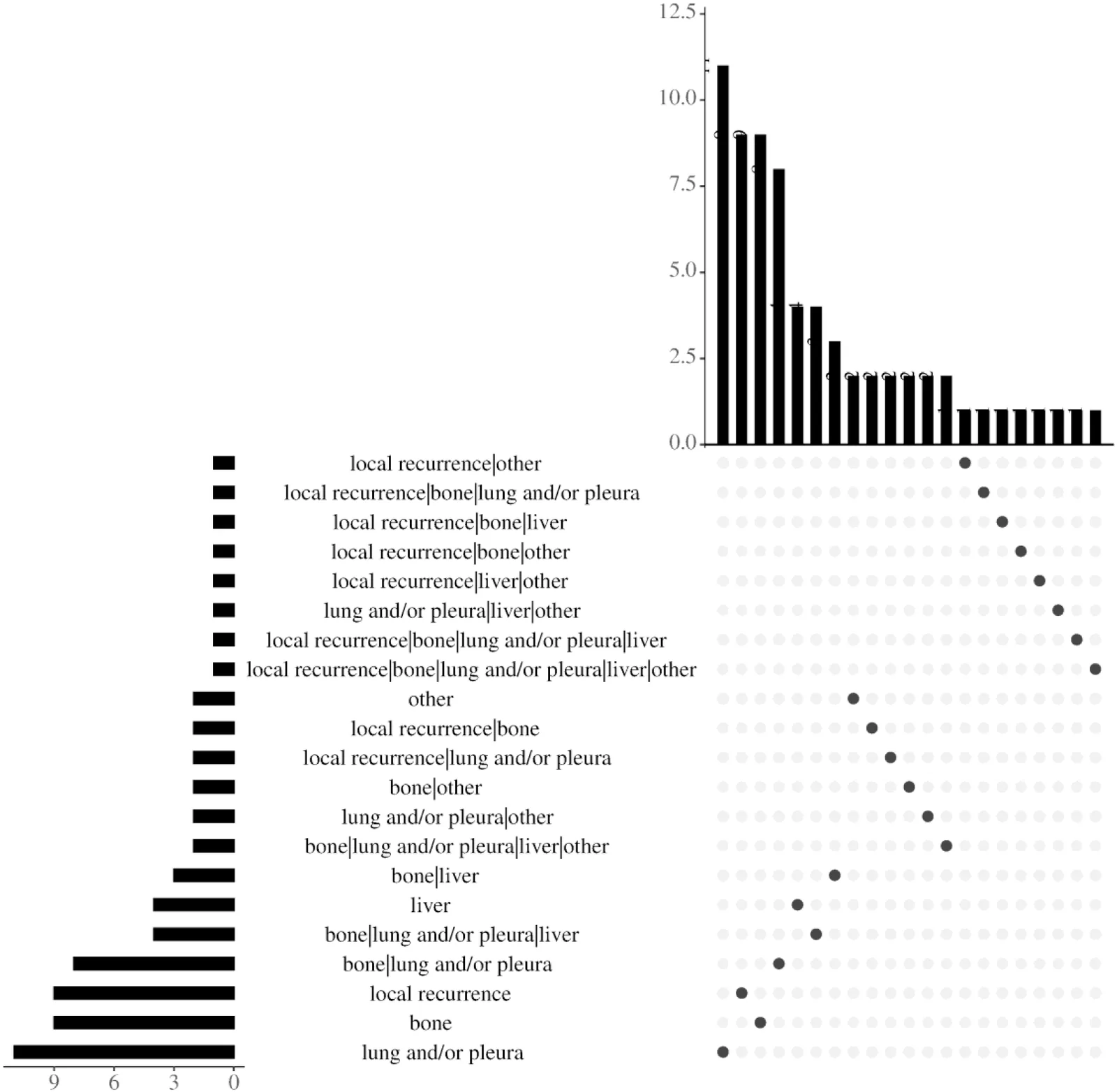
UpSet plot illustrating the distribution of recurrence sites in breast cancer patients.

### Interobserver agreement

To assess interobserver reproducibility, kappa coefficient was calculated for each of the 14 MRI features individually.

Interobserver agreement was substantial for all of features, FGT (κ = 0.697, 95% CI: 0.587 ~ 0.789), BPE (κ = 0.763, 95% CI: 0.656 ~ 0.845), tumor size (κ = 0.737, 95% CI: 0.628 ~ 0.823), tumor type (κ = 0.724, 95% CI: 0.614 ~ 0.812), tumor shape (κ = 0.750, 95% CI: 0.642 ~ 0.834), tumor margin (κ = 0.684, 95% CI: 0.573 ~ 0.778), peritumoral oedema (κ = 0.829, 95% CI: 0.729 ~ 0.897), adjacent vessel sign (κ = 0.734, 95% CI: 0.628 ~ 0.823), peripheral tissue invasion (κ = 0. 921, 95% CI: 0.838 ~ 0.963), duct dilatation (κ = 0.855, 95% CI: 0.759 ~ 0.917), nipple-areola complex invasion (κ = 0.868, 95% CI: 0.774 ~ 0.927), enhancement pattern (κ = 0.734, 95% CI: 0.628 ~ 0.823), TIC (κ = 0.882, 95% CI: 0.790 ~ 0.936) and ALNM (κ = 0.763, 95% CI: 0.656 ~ 0.845).

### Patient matching and cohort characteristics

Using a 1:2 PSM ratio, 68 patients with recurrence were matched to 136 patients without recurrence, yielding 204 matched patients, who were then randomly allocated to a training set (n = 142) and a validation set (n = 62). No significant differences were observed in any MRI feature between the two sets (all P > 0.05) (**Supplementary Table 1**).

SMD for all baseline characteristics before and after PSM are presented in **Supplementary Table 2**. After PSM, the matching variables, age and family history of BC, achieved adequate balance (SMD < 0.1), with SMD values of 0.043 and 0.050, respectively. However, type of surgery remained imbalanced after matching (SMD = 0.147), exceeding the conventional threshold of 0.1. To address this residual confounding, this variable was entered into the multivariable model as a covariate. For the non-matching variables, SMD values remained largely unchanged.

### Independent predictive factors for recurrence

With 68 recurrence events and 14 candidate variables considered in the univariate analysis, the EPV for the full candidate set was 68/14 ≈ 4.9, which is slightly below the commonly recommended minimum threshold of 5–10 events per variable.

Univariate and multivariate logistic regression analyses were performed in the training set, and the results are presented in **Table 2**. In the univariate analysis, tumor size (>5 cm), peritumoral edema, adjacent vessel sign, peripheral tissue invasion, nipple-areola complex invasion, enhancement pattern, and ALNM were significantly associated with recurrence. No significant associations were observed for FGT, BPE, tumor shape, margin, tumor type, duct dilatation, or TIC type. In the multivariate logistic regression analysis, tumor size (>5 cm), adjacent vessel sign, and ALNM were identified as independent predictors of recurrence.

**Table 2:** Logistic regression analysis in the training cohort.

MRI features	Univariate analysis		Multivariate analysis	
	OR (95% CI)	*P*	OR (95% CI)	*P*
Tumor size				
≤5cm	Ref		Ref	
>5cm	7.628 (3.492-16.660)	<0.001	2.832 (1.030-7.782)	0.044
Amount of fibroglandular tissue		0.284		
Almost entirely fatty	Ref			
Scattered	1.613 (0.565-4.605)	0.372		
Heterogeneous	0.743 (0.245-2.250)	0.599		
Extreme	0.833 (0.209-3.321)	0.796		
Background parenchymal enhancement		0.169		
Non/minimal	Ref			
Mild	0.485 (0.196-1.200)	0.118		
Moderate	0.523 (0.130-2.101)	0.361		
Marked	0.174 (0.021-1.453)	0.106		
Tumor type		0.633		
Single mass	Ref			
Nonmass enhancement	1.279 (0.565-2.898)	0.555		
Multi-mass	1.607 (0.546-4.732)	0.389		
Tumor shape	0.765 (0.365-1.604)	0.478		
Tumor margin	0.638 (0.318-1.283)	0.207		
Peritumoral edema	2.407 (1.180-4.912)	0.016		
Adjacent vessel sign	4.427 (2.075-9.448)	<0.001	2.704 (1.068-6.845)	0.036
Duct dilatation	1.103 (0.403-3.017)	0.849		
Peripheral tissue invasion	4.033 (1.838-8.852)	0.001		
Nipple-areola complex invasion	3.425 (1.234-9.504)	0.018		
Enhancement pattern	2.311 (1.137-4.698)	0.021		
Time-signal intensity curve		0.160		
Persistent	Ref			
Plateau	0.298 (0.077-1.156)	0.080		
Wash out	0.693 (0.232-2.069)	0.511		
Axillary lymph node metastasis	7.500 (3.320-16.994)	<0.001	2.860 (1.005-8.136)	0.049

cm, centimetre; OR, odds ratio.

The final multivariable logistic regression model was specified as follows: Logit(P) = −2.977 + 1.041 × (Tumor size >5 cm) + 1.051 × (ALNM) +0.995 × (Adjacent vessel sign).

### Development and performance of the nomogram

A nomogram incorporating all identified independent predictors was constructed to predict the individualized probability of postoperative recurrence (**Figure 3**). In the nomogram, each predictor is assigned a score on the point scale; the total score, obtained by summing individual scores, corresponds to a predicted recurrence risk on the lower axis. According to this scoring system, higher total scores were associated with a greater probability of recurrence (**Figure 4**).

**Figure 3:**
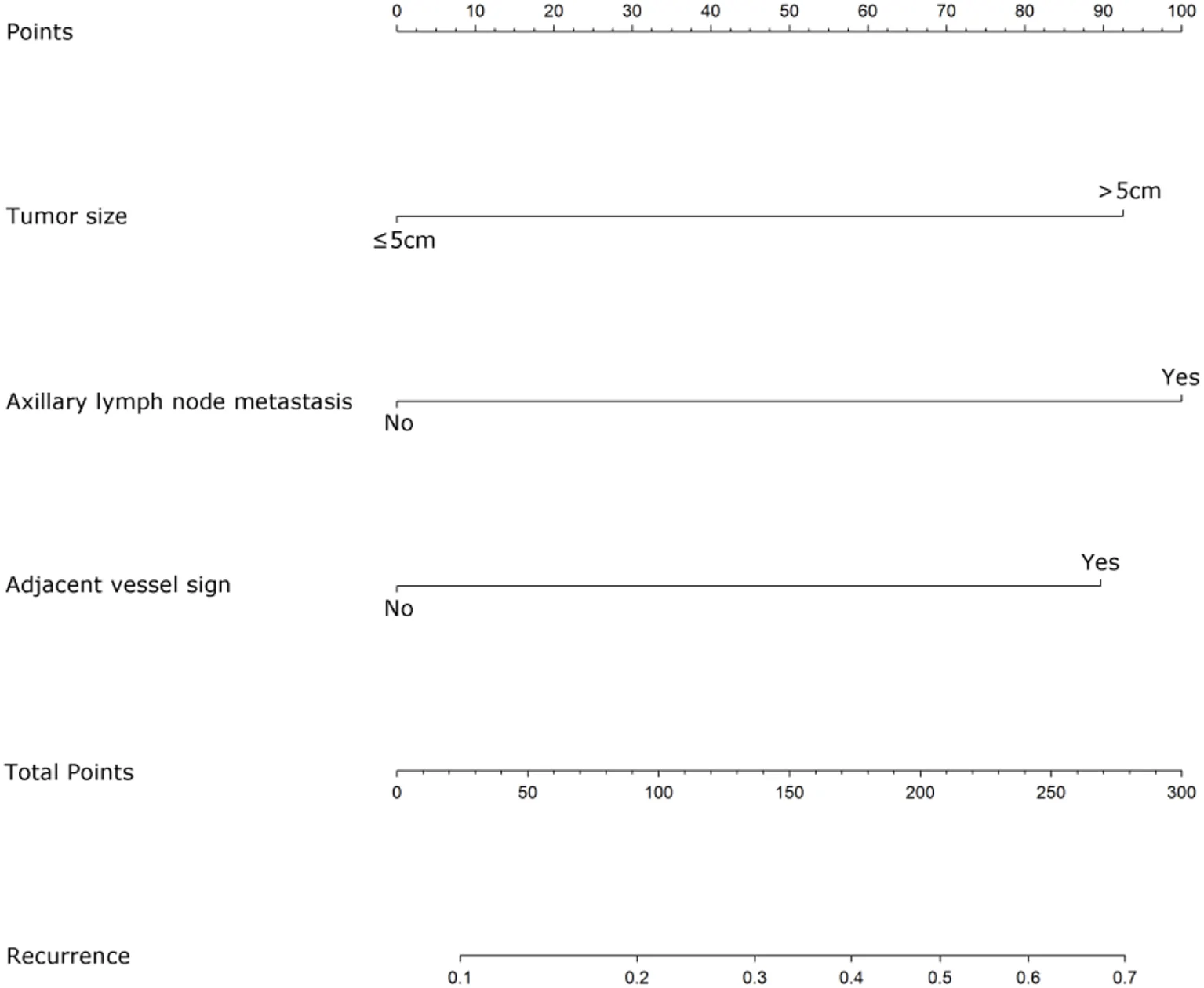
Nomogram for predicting the probability of recurrence in patients with breast cancer.

**Figure 4:**
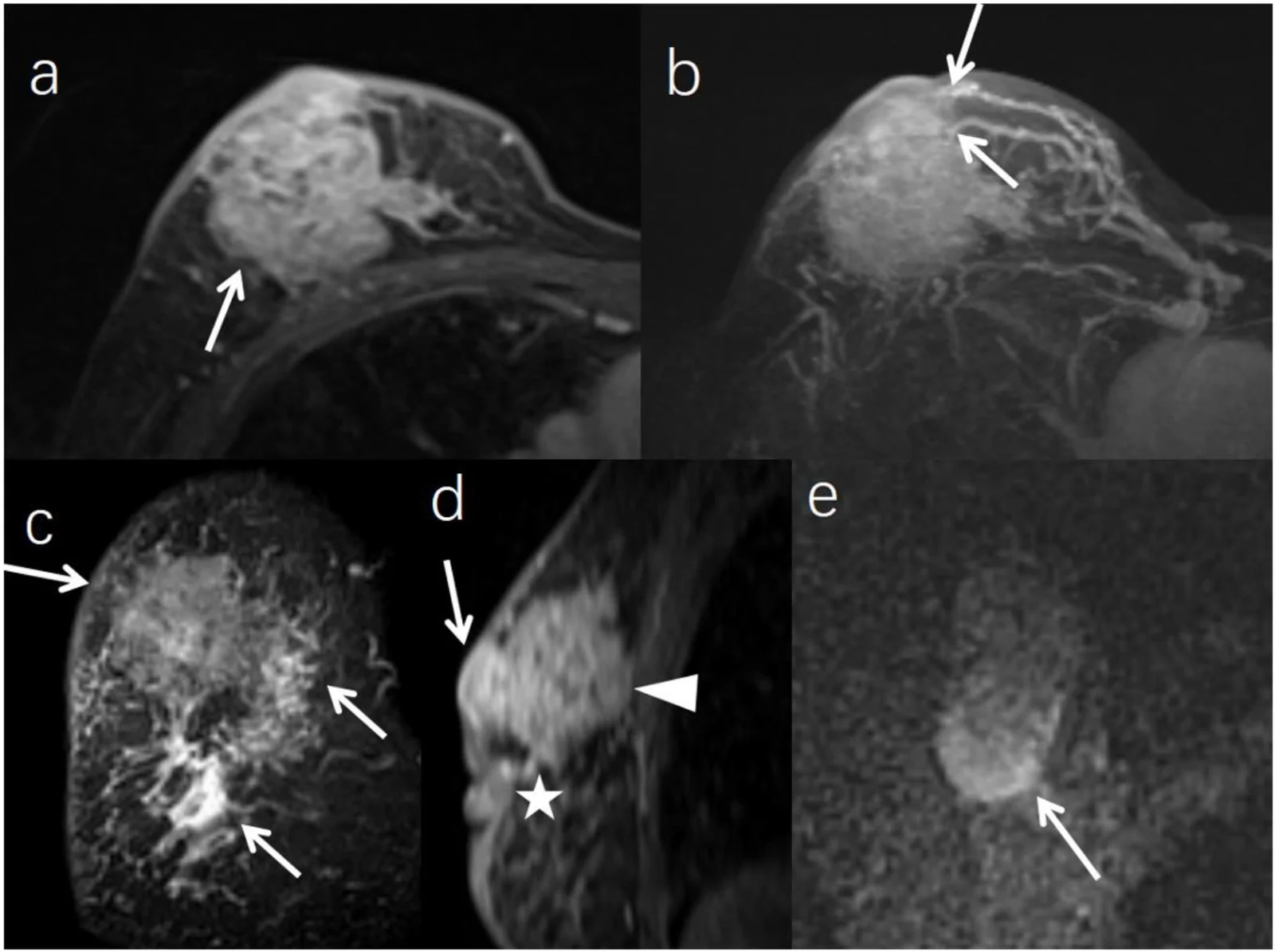
A 74-year-old woman classified as high risk by the predictive model. **(a)** Axial fat-suppressed contrast-enhanced T1-weighted MR image shows a 4.5-cm irregular enhancing mass (arrow) in the right breast. **(b)** Three-dimensional maximum intensity projection image shows vessels entering the lesion (arrows). **(c)** Coronal fat-suppressed T2-weighted MR image shows the mass with peritumoral edema (arrows). **(d)** Sagittal fat-suppressed contrast-enhanced T1-weighted MR image shows tumor invasion of the skin (arrow), chest wall (arrowhead), and nipple-areola complex (star). **(e)** Coronal fat-suppressed T2-weighted MR image shows an enlarged lymph node (arrow), which was pathologically confirmed as lymph node metastasis. After radical mastectomy, the final pathological examination confirmed invasive ductal carcinoma. The patient was categorized as high risk for recurrence and had evidence of lung and brain metastases at 24 months of follow-up.

The nomogram showed good discriminative ability, with an AUC of 0.809 (95% CI: 0.736–0.881) in the training set and 0.797 (95% CI: 0.684–0.910) in the validation set (**Figure 5**). After bootstrap correction with 1000 iterations, the optimism-corrected AUC was 0.798 (95% CI 0.724-0.876), with a mean optimism of 0.011, indicating negligible overfitting and good discriminative performance.

**Figure 5:**
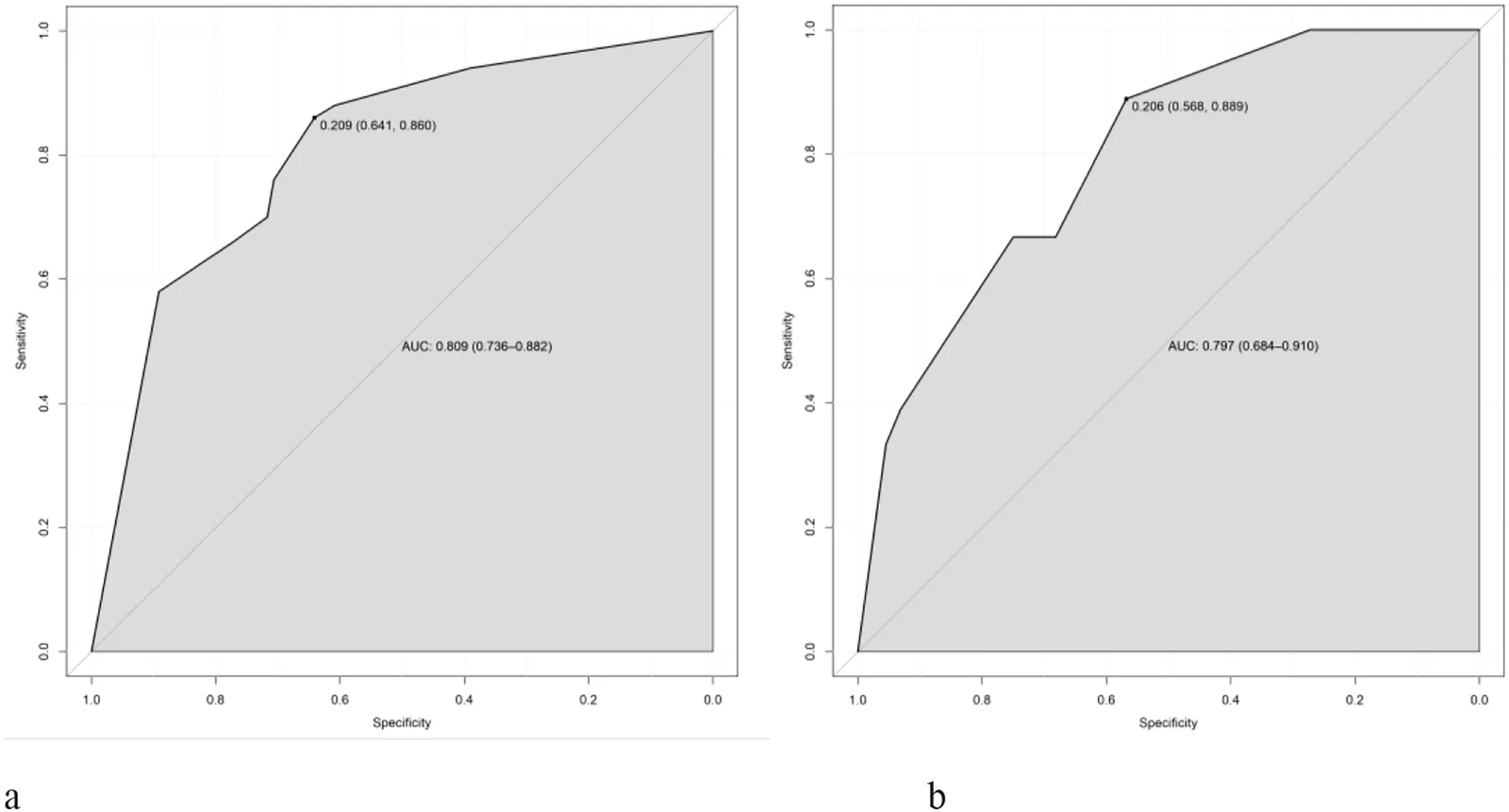
Receiver operating characteristic (ROC) curves of the nomogram in the **(a)** training and **(b)** validation cohorts. The area under the curve (AUC) was 0.809 (95% confidence interval: 0.736–0.882). The optimal cut-off value of 0.209 was determined based on the maximum Youden index, corresponding to a sensitivity of 0.641 and a specificity of 0.860 in the training cohort. The area under the curve (AUC) was 0.797 (95% confidence interval: 0.684–0.910). The optimal cut-off value of 0.206 was determined based on the maximum Youden index, corresponding to a sensitivity of 0.568 and a specificity of 0.889 in the validation cohort.

Calibration performance was evaluated separately in the training and validation cohorts. (**Figure 6**) In the training cohort, the calibration intercept was 0.00 (95% CI: −0.41 to 0.40) and the calibration slope was 1.00 (95% CI: 0.67 to 1.37), both approximating the ideal values of 0 and 1, respectively. The MAE between predicted and observed probabilities was 0.087. The Hosmer–Lemeshow test indicated acceptable calibration (χ² = 5.88, P = 0.117). In the validation cohort, the calibration intercept was −0.138 (95% CI: −0.766 to 0.454) and the calibration slope was 1.100 (95% CI: 0.483 to 1.846). The MAE was 0.070. The Hosmer–Lemeshow test also supported satisfactory calibration (χ² = 6.305, P = 0.098). Collectively, these findings demonstrate satisfactory calibration of the nomogram across both cohorts, although the near-ideal estimates in the training set should be interpreted with caution given the limited sample size.

**Figure 6:**
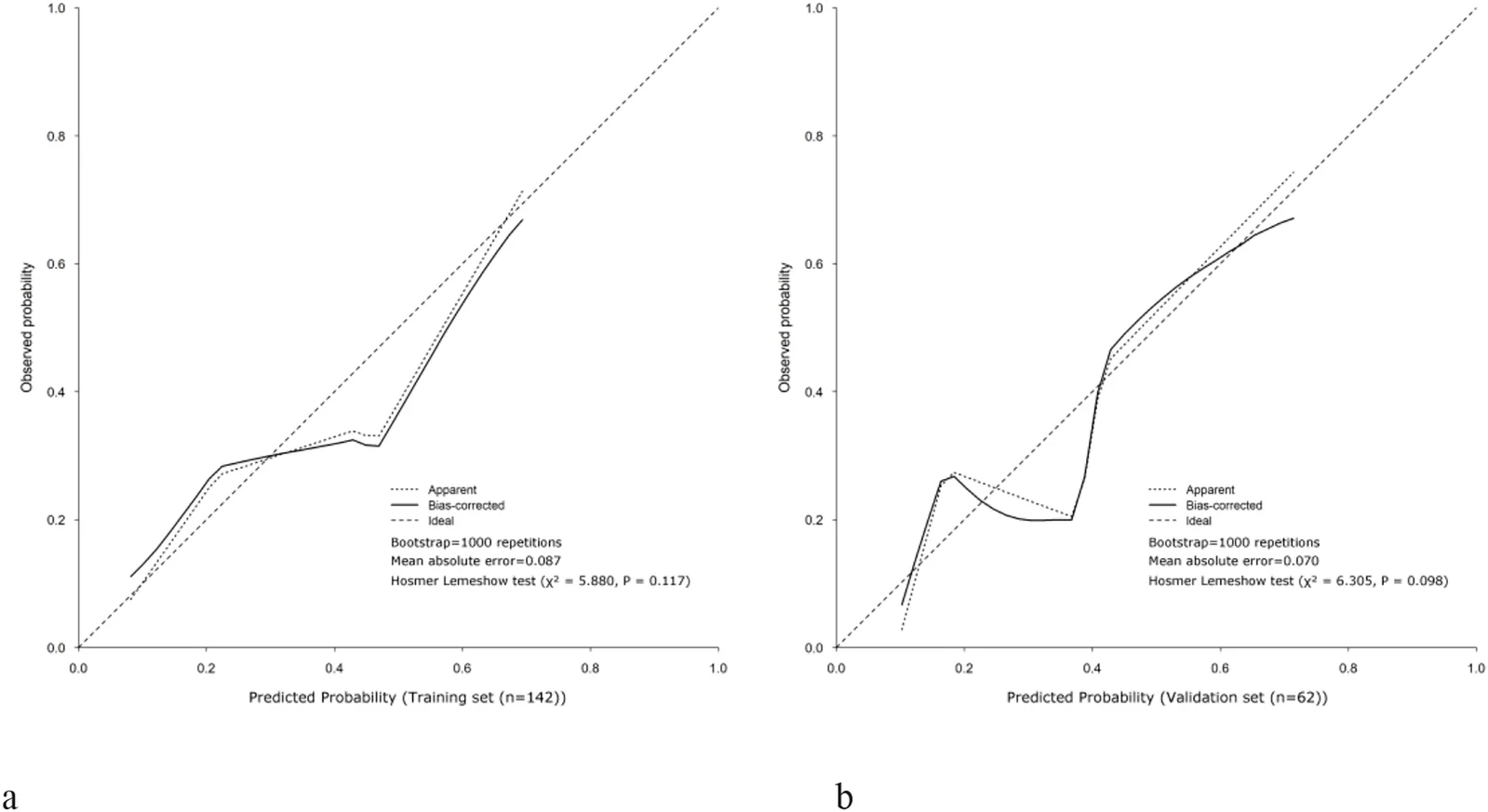
Calibration curves of the nomogram in the **(a)** training cohort (n = 142) and **(b)** validation cohort (n = 62). The x-axis represents the predicted probability of recurrence, and the y-axis represents the observed probability. The 45-degree dashed diagonal line represents perfect calibration. The dashed line indicates apparent performance, and the solid line indicates bias-corrected performance after 1000 bootstrap iterations. Apparent (dashed line) and bias-corrected (solid line) curves closely follow the ideal line in both cohorts. The mean absolute error (MAE) between the predicted and observed probabilities was 0.087 for the training cohort and 0.070 for the validation cohort. The Hosmer-Lemeshow goodness-of-fit test showed no significant lack of fit in either cohort (training: χ² = 5.880, P = 0.117; validation: χ² = 6.830, P = 0.098).

DCA confirmed that the nomogram provided a positive net benefit across threshold probabilities ranging from approximately 10% to 70%, demonstrating favorable clinical utility (**Figure 7**). In clinical contexts, this threshold range aligns with​ the risk level at which clinicians would typically consider intensified surveillance (e.g., more frequent imaging or biomarker monitoring) or adjuvant treatment intensification. Relative to​ the “treat all” and “treat none” strategies, the nomogram achieved superior net benefit across this range, underscoring​ its potential utility in guiding personalized clinical decisions.

**Figure 7:**
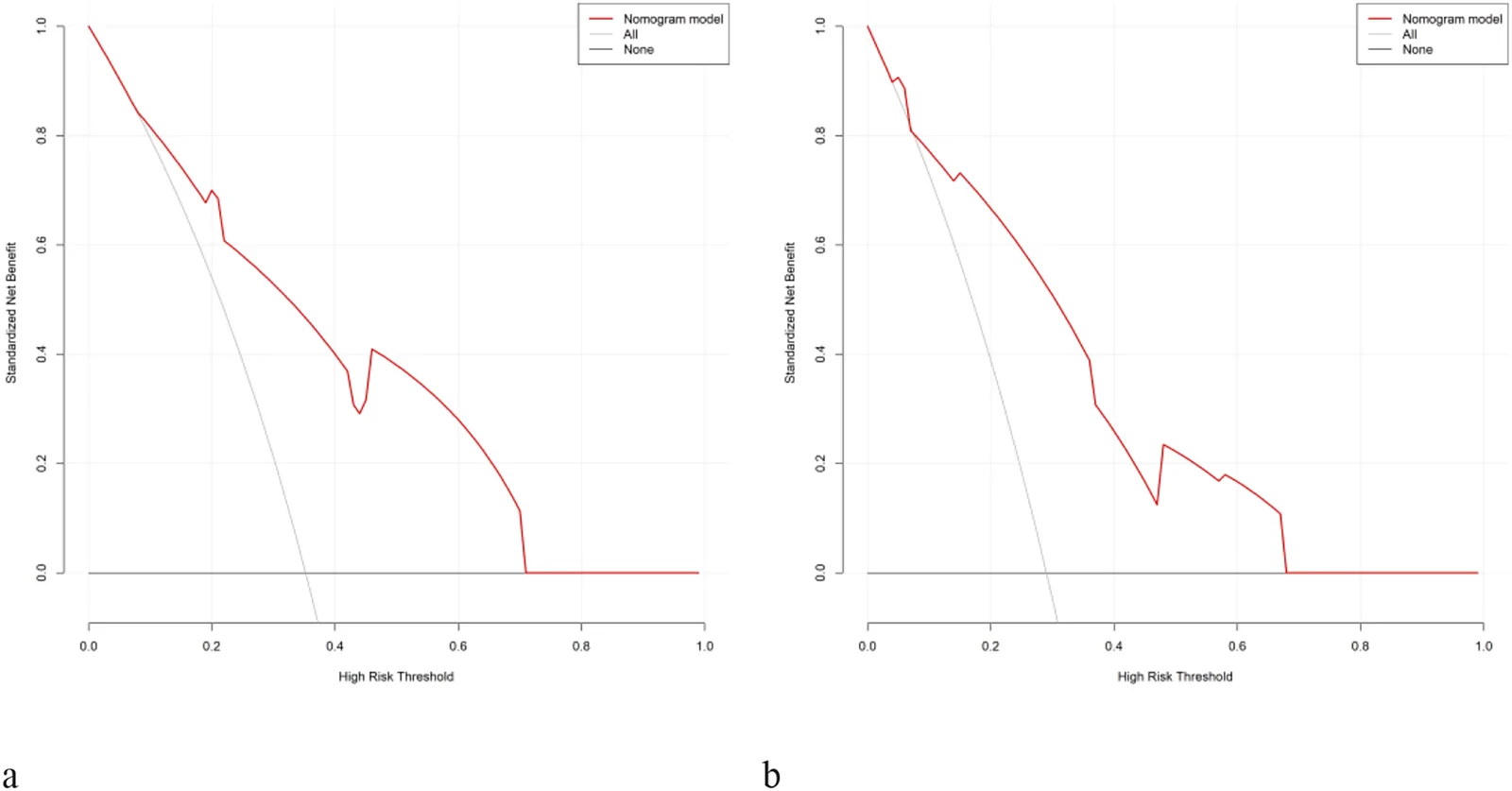
Decision curve analysis (DCA) for the nomogram in the **(a)** training and **(b)** validation cohorts. The x-axis represents the threshold probability, and the y-axis represents the net benefit. The red line represents the nomogram; the gray dashed line represents the “All” strategy (all patients treated); the black horizontal line represents the “None” strategy (no patients treated). The nomogram provided a positive net benefit across threshold probabilities ranging from approximately 10% to 70%. This threshold range corresponds to clinically relevant risk levels at which clinicians would typically consider intensified surveillance or adjuvant treatment intensification. Compared with the “treat-all” and “treat-none” strategies, the nomogram achieved superior net benefit across this range, supporting its potential utility in guiding personalized clinical decisions.

## Discussion

Although the mortality rate of BC has decreased, postoperative recurrence remains a significant clinical challenge. In this study, a preoperative MRI-based nomogram incorporating semantic features was developed and internally validated to predict postoperative recurrence in BC. The model demonstrated robust discriminative performance (optimism-corrected AUC = 0.789) and satisfactory calibration in this single-center cohort. These findings highlight DCE-MRI as a valuable non-invasive tool that can assist clinicians in assessing recurrence probability, thereby facilitating personalized treatment planning prior to definitive therapy. Nevertheless, the generalizability of this model warrants further validation in external, multicenter cohorts. In recent years, several predictive models for distant metastasis in BC have been reported. Ma W et al. constructed a fusion model integrating clinicopathological and MRI features, which demonstrated strong predictive performance for distant metastasis, with an AUC of 0.854 (12). Yao Y et al. established a multimodal deep learning model that combined pathological, clinical, and gene expression data to assess recurrence and metastasis risk (15). Similarly, Chae EY et al. developed a prognostic model based on preoperative breast MRI and clinicopathological features to predict recurrence in women aged ≤35 years (16). While these prior models have demonstrated predictive value, many of these approaches, particularly radiomics-based or deep learning models, require complex post-processing, specialized software, or proprietary algorithms, which substantially limits their accessibility in routine clinical practice. Moreover, most relied predominantly on clinicopathological and postoperative pathological variables, with limited adjustment for potential confounders. BC frequently exhibits genetic predisposition, and a family history of BC typically classifies patients as high-risk. Moreover, factors such as younger age and breast-conserving surgery are established independent risk factors for recurrence (17). To mitigate the confounding effects of these factors, age, family history of BC, and type of surgery were incorporated as matching variables in PSM in this study, thereby enhancing comparability between recurrence and non-recurrence groups. Compared with these previously reported models, this model offers several distinct advantages. First, the model incorporates a comprehensive set of preoperative MRI features, which may more accurately capture the macroscopic, microscopic, and heterogeneous characteristics of the tumor, thereby facilitating a deeper understanding of the value of specific MRI features in predicting recurrence risk. Importantly, these semantic MRI features can be visually assessed on routine clinical images without requiring specialized software or post-processing, making the model more suitable for real-world clinical implementation, particularly in resource-constrained​ settings. Second, PSM was used to mitigate potential biases​ by balancing baseline clinical characteristics, thereby allowing for the robust isolation​ of the independent prognostic value of MRI features.

Our results identified tumor size (>5 cm) and ALNM as independent risk factors for recurrence, which aligns with previously reported evidence. Tumor size and ALNM are well-established prognostic factors that reflect disease progression; larger tumors are generally associated with more aggressive biological behavior. Delpech et al. confirmed that both T and N stages are independently associated with bone-only metastasis in BC (18). Similarly, Ma W et al. demonstrated a correlation between ALNM and distant metastasis (12). In another study, Chae EY et al. identified tumor size as an independent predictor of recurrence in young women with BC (16). Collectively, these consistent findings underscore that tumor burden and lymph node involvement remain key drivers of postoperative recurrence, supporting the rationale for incorporating these variables into preoperative risk stratification tools. In this context, semantic MRI features offer non-invasive insights into the tumor microenvironment and angiogenic activity, thereby providing incremental predictive value.

Angiogenesis serves as a critical early step to support oxygen supply during the metastatic cascade (19). Angiogenesis plays a crucial role in BC recurrence (20); increased peritumoral vascularization facilitates tumor cell enter into the peripheral bloodstream, promoting cancer spread to distant sites and increasing the likelihood of early recurrence (21). The adjacent vessel sign, an important imaging feature reflecting tumor vascularity, is closely related to the tumor microenvironment. In this study, the adjacent vessel sign was identified as an independent risk factor for recurrence, consistent with the findings of Han et al., who demonstrated that increased peritumoral vascularity is associated with malignancy, lymph node metastasis, and an increased risk of distant metastasis (22). Furthermore, peritumoral edema was associated with recurrence in this study, although it did not serve as an independent predictor. In contrast,​ Chae EY et al. identified peritumoral edema as an independent predictor of recurrence in young women with BC (16), which may be attributed to differences in study populations or covariate adjustment. Biologically, peritumoral edema may be related to tumor neovascularization and the expression of vascular endothelial growth factor and tumor-related cytokines (23). More fundamentally, peritumoral edema has been associated with multiple genes contributing to treatment resistance and tumor recurrence (24). Of note, the adjacent vessel sign and peritumoral edema are not mutually independent imaging features. From a pathophysiological perspective, active angiogenesis drives both increased vascularity, which constitutes the morphological basis of the adjacent vessel sign, and increased vascular permeability, which underlies peritumoral edema (25). These two features therefore reflect different facets of the same biological process, and their concurrent retention and synergistic predictive value in the present model are biologically coherent.

Among the other MRI features evaluated, peripheral tissue invasion, nipple-areola complex invasion, and the assessed tumor-to-nipple distance​ remain important considerations in determining eligibility for nipple-sparing mastectomy (26). In this study, both peripheral tissue invasion and nipple-areola complex invasion were significantly associated with postoperative recurrence. Previous studies have shown that a shorter tumor-to-nipple distance increases the risk of locoregional recurrence following breast-conserving surgery (27). Nipple-areola complex invasion is also associated with a higher probability of major lactiferous duct invasion, which may facilitate tumor cell migration to the periphery. Moreover, BC exhibiting peripheral tissue invasion is generally classified as locally advanced and is associated with poorer prognosis, as well as higher rates of locoregional and distant metastasis. For patients with MRI-detected nipple-areola complex invasion or peripheral tissue invasion, surgeons may consider wider surgical margins or recommend neoadjuvant therapy to downstage the tumor prior to surgery. These features are valuable for preoperative decision-making. However, the independent prognostic value of these features for predicting recurrence warrants further validation in larger cohorts.

Several limitations of this study should be acknowledged. First, the single-center retrospective design introduces potential selection and information biases, thereby limiting the external validity of the study’s results. Second, although PSM was applied to balance baseline characteristics, only three variables (age, family history, and type of surgery) were included in the matching algorithm, attributable to​ the limited number of recurrence events (n = 68) and to avoid over-adjustment for factors closely correlated with MRI features. Variables such as tumor stage, grade, and molecular subtype were intentionally not included in the matching algorithm, as these factors may lie on the causal pathway between imaging phenotypes and recurrence. Consequently, residual confounding from these unadjusted variables cannot be entirely excluded, and the results should be interpreted with caution. Third, competing risks, such as death without recurrence, were not formally modeled in this analysis. In the present cohort, the number of such events was small and unlikely to materially affect the estimates. Nevertheless, in long-term follow-up studies, competing events may influence the cumulative incidence of recurrence. Future studies with larger sample sizes should consider using competing risk regression (e.g., the Fine-Gray model) to account for this. Fourth, although bootstrap internal validation with 1000 iterations suggested minimal overfitting (optimism = 0.011), the possibility of overfitting cannot be entirely excluded, particularly given the modest sample size of the validation cohort (n = 62), which may affect the precision of performance estimates. Additionally, the calibration slope and intercept were very close to their ideal values (1.000 and −0.000, respectively). While these findings suggest satisfactory calibration, such near-perfect estimates may reflect sample-specific variation or over-smoothing rather than true generalizable performance. Therefore, these calibration metrics should be interpreted with caution, and external validation in larger independent cohorts is required to confirm the robustness of the model’s calibration and generalizability. Future research should prioritize multicenter external validation, integration of radiomics or deep learning for automated feature extraction, and combination of MRI with molecular markers to improve risk stratification.

## Conclusion

In conclusion, a preoperative DCE-MRI-based nomogram incorporating the adjacent vessel sign, tumor size, and axillary lymph node metastasis was developed and internally validated for predicting postoperative recurrence in BC. By generating individualized recurrence risk estimates prior to treatment using semantic MRI features visually assessable on routine clinical images, this nomogram holds promise for guiding clinical decision-making and optimizing treatment stratification. External validation in large, multicenter prospective cohorts is warranted to confirm its clinical utility.

## Data Availability

The data analyzed in this study is subject to the following licenses/restrictions: Data are available from the corresponding author upon reasonable request, subject to ethical restrictions. Requests to access these datasets should be directed to jeanbujun@163.com.
