# Supplementary material for: A preoperative nomogram incorporating semantic MRI features for predicting breast cancer recurrence: a propensity score-matched cohort study
Source: Front Oncol. 2026 Sep 24;16:1886709. doi: 10.3389/fonc.2026.1886709 (PMC13645599; doi:10.3389/fonc.2026.1886709)
Supplement: Supplementary file 1 [file Table1.docx]

Tabel 1 Characteristics of patients with breast cancer in the training and validation sets

| **MRI features** | **Total**  **(N=204)** | **Training set**  **(N=142)** | **Validation set**  **(N=62)** | **χ^2^** | ***P* value** |
| --- | --- | --- | --- | --- | --- |
| Tumor size |  |  |  | 2.557 | 0.279 |
| <2 cm | 77 | 58 | 19 |  |  |
| 2 -5 cm | 94 | 64 | 30 |  |  |
| >5 cm | 33 | 20 | 13 |  |  |
| Amount of fibroglandular tissue |  |  |  | 3.162 | 0.367 |
| Almost entirely fatty | 34 | 21 | 13 |  |  |
| Scattered | 84 | 56 | 28 |  |  |
| Heterogeneous | 65 | 48 | 17 |  |  |
| Extreme | 21 | 17 | 4 |  |  |
| Background parenchymal enhancement |  |  |  | 2.882 | 0.410 |
| Non - minimal | 138 | 91 | 47 |  |  |
| Mild | 41 | 31 | 10 |  |  |
| Moderate | 14 | 11 | 3 |  |  |
| Marked | 11 | 9 | 2 |  |  |
| Tumor type |  |  |  | 3.549 | 0.170 |
| Mass | 126 | 92 | 34 |  |  |
| Non-mass enhancement | 49 | 34 | 15 |  |  |
| Multifocal | 29 | 16 | 13 |  |  |
| Tumor shape |  |  |  | 0.420 | 0.517 |
| Regular | 59 | 43 | 16 |  |  |
| Irregular | 145 | 99 | 46 |  |  |
| Tumor margin |  |  |  | 0.025 | 0.874 |
| Circumscribed | 107 | 75 | 32 |  |  |
| Not circumscribed | 97 | 67 | 30 |  |  |
| Peritumoral edema |  |  |  | 1.545 | 0.214 |
| Absent | 120 | 90 | 30 |  |  |
| Present | 84 | 52 | 32 |  |  |
| Adjacent vessel sign |  |  |  | 0.048 | 0.826 |
| Absent | 89 | 66 | 23 |  |  |
| Present | 115 | 76 | 39 |  |  |
| Duct dilatation |  |  |  | 0.442 | 0.802 |
| Absent | 183 | 127 | 56 |  |  |
| Present | 21 | 15 | 6 |  |  |
| Peripheral tissue invasion |  |  |  | 3.029 | 0.082 |
| Absent | 166 | 120 | 46 |  |  |
| Present | 38 | 22 | 16 |  |  |
| Nipple-areola complex invasion |  |  |  | 2.694 | 0.101 |
| Absent | 171 | 123 | 48 |  |  |
| Present | 33 | 19 | 14 |  |  |
| Enhancement pattern |  |  |  | 2.589 | 0.108 |
| Homogeneous | 93 | 70 | 23 |  |  |
| Heterogeneous | 111 | 72 | 39 |  |  |
| Time-signal intensity curve |  |  |  | 0.371 | 0.831 |
| Persistent | 20 | 15 | 5 |  |  |
| Plateau | 43 | 29 | 14 |  |  |
| Wash out | 141 | 98 | 43 |  |  |
| Axillary lymph node metastasis |  |  |  | 0.004 | 0.948 |
| Absent | 98 | 68 | 30 |  |  |
| Present | 106 | 74 | 32 |  |  |

cm: centimeter

### Table 2. Standardized Mean Differences Before and After Propensity Score Matching

| **Variables** | **Pre-Matching SMD** | **Post-Matching SMD** |
| --- | --- | --- |
| **Matching variables** |  |  |
| Age | 0.068 | 0.043 |
| Family history of breast cancer | 0.065 | 0.050 |
| Type of surgery | 0.319 | 0.147 |
| **Non-matching variables** |  |  |
| FGT | 0.168 | 0.178 |
| BPE | 0.317 | 0.304 |
| Tumor size | 0.726 | 0.716 |
| tumor type | 0.196 | 0.229 |
| tumor shape | 0.091 | 0.256 |
| tumor margin | 0.187 | 0.193 |
| peritumoral oedema | 0.320 | 0.315 |
| adjacent vessel sign | 0.511 | 0.446 |
| duct dilatation | 0.095 | 0.175 |
| peripheral tissue invasion | 0.456 | 0.457 |
| nipple-areola complex invasion | 0.447 | 0.460 |
| Enhancement pattern | 0.361 | 0.363 |
| time-signal intensity curve type | 0.182 | 0.243 |
| axillary lymph node metastasis | 0.541 | 0.235 |
